# Effect of land use and soil organic matter quality on the structure and function of microbial communities in pastoral soils: Implications for disease suppression

**DOI:** 10.1371/journal.pone.0196581

**Published:** 2018-05-07

**Authors:** Bryony E. A. Dignam, Maureen O’Callaghan, Leo M. Condron, George A. Kowalchuk, Joy D. Van Nostrand, Jizhong Zhou, Steven A. Wakelin

**Affiliations:** 1 Bio-protection Research Centre, Lincoln University, Lincoln, Christchurch, New Zealand; 2 Soil Biology, Farm Systems & Environment, AgResearch Ltd, Lincoln, Christchurch, New Zealand; 3 Ecology and Biodiversity, Institute of Environmental Biology, Utrecht University, Utrecht, The Netherlands; 4 Institute for Environmental Genomics, University of Oklahoma, Norman, Oklahoma, United States of America; 5 Earth Sciences Division, Lawrence Berkeley National Laboratory, Berkeley, California, United States of America; 6 State Key Joint Laboratory of Environment Simulation and Pollution Control, School of Environment, Tsinghua University, Beijing, China; 7 Scion Research Ltd, Christchurch, New Zealand; Universite Paris-Sud, FRANCE

## Abstract

Cropping soils vary in extent of natural suppression of soil-borne plant diseases. However, it is unknown whether similar variation occurs across pastoral agricultural systems. We examined soil microbial community properties known to be associated with disease suppression across 50 pastoral fields varying in management intensity. The composition and abundance of the disease-suppressive community were assessed from both taxonomic and functional perspectives. *Pseudomonas* bacteria were selected as a general taxonomic indicator of disease suppressive potential, while genes associated with the biosynthesis of a suite of secondary metabolites provided functional markers (GeoChip 5.0 microarray analysis). The composition of both the *Pseudomonas* communities and disease suppressive functional genes were responsive to land use. Underlying soil properties explained 37% of the variation in *Pseudomonas* community structure and up to 61% of the variation in the abundance of disease suppressive functional genes. Notably, measures of soil organic matter quality, C:P ratio, and aromaticity of the dissolved organic matter content (carbon recalcitrance), influenced both the taxonomic and functional disease suppressive potential of the pasture soils. Our results suggest that key components of the soil microbial community may be managed on-farm to enhance disease suppression and plant productivity.

## Introduction

Agricultural grasslands are an extensive form of land use and are a key resource in terms of biodiversity and ecosystems services [1]. As global demand for livestock production (food and fiber) increases, pastoral agriculture is undergoing intensification. This is driven by increased inputs of fertilizers and water, alteration of the botanical composition, and shifts in grazing management. Collectively, these lead to new abiotic and biotic environments within which pasture diseases may develop [2].

Diverse and dynamic soil-borne pathogen complexes develop in pastoral agricultural systems [2]. These constrain potential primary productivity and reduce the efficiency (plant utilization per unit growth) of water and nutrient utilization [3]. Due to the complexity of disease symptoms, and their typical manifestation below-ground, yield losses directly attributable to soil-borne pathogens in pastures are often unrecognized and greatly underestimated. Thus, across agricultural systems, soil-borne diseases remain a costly yet intractable management challenge [4].

In disease suppressive soils, consortia of microbial taxa protect susceptible plant hosts from disease caused by soil-borne pathogens [5–9]. This phenomenon is driven either by the competitive activity of the total soil microbiota (general suppression), or the antagonistic potential of an individual or specific group of microorganisms (specific suppression; [10]).

Management of the soil microbial community towards inducing or enhancing disease suppression presents an emerging, and potentially more enduring, approach to disease control in agricultural systems [11]. Managing or ‘engineering’ the soil microbiome in this way may prove to be particularly important in pastoral grasslands where the control of diverse pathogens is complicated by multi-plant-multi-pathogen interactions [2, 12]. To exploit the natural processes that lead to enhanced disease suppression in pastures, and to support farmers in managing pastoral systems towards a more suppressive state, it is important to develop understanding of the occurrence of suppressive microbiota in the field and how these vary spatially, temporally, and with farm management practices.

Members of a diverse range of bacterial genera have been identified as ‘disease suppressive bacteria’. These include *Agrobacterium*, *Arthrobacter*, *Azotobacter*, *Bacillus*, *Burkholderia*, *Collimonas*, *Pantoea*, *Pseudomonas*, *Serratia*, *Stenotrophomonas*, and *Streptomyces* spp. [13]. Of these, *Pseudomonas* spp. have widely been employed as model organisms for the investigation of biocontrol mechanisms [14], as their role in disease suppression has been consistently and independently established across studies [5, 6].

This study surveyed the impacts of land use, biogeography, environment, and soil type on the composition and abundance of the microbial community in pastoral soils. The community was examined with a specific focus on both taxonomic and functional characteristics related to putative disease suppressive potential. Phylogenetic community analyses targeted *Pseudomonas* spp. as a general taxonomic indicator of disease suppressive potential. There is increasing evidence that the bacterial community and specific taxa or functional groups within this are differentially influenced by alterations in soil properties that result from varying farm management practices in both arable (for example, [15]) and grassland (for example, [16]) systems. As such, assessments of the total bacterial community were conducted concomitantly to enable findings associated with *Pseudomonas* to be interpretable as either ‘specific’ to these taxa, or ‘general’ as part of changes in the wider bacterial community. Phylogenetic community analyses was coupled with functional gene microarray analysis (GeoChip 5.0) targeting functional genes putatively linked with suppressive activity: nutrient competition (siderophore production; [17]), hyperparasitism (fungal cell wall degradation; [18, 19]), and antibiosis (secondary metabolite biosynthesis; [13]). While this ecologically based study did not aim to define associative links between soil microbial communities and plant disease incidence, a range of soil properties were assessed to provide fundamental understanding of how *Pseudomonas* communities, and functional genes putatively associated with disease suppression are being significantly modified in pastoral ecosystems.

We hypothesised that: (1) the *Pseudomonas* community structure would be under different selective pressure compared with the wider bacterial community; (2) the abundance of putative disease suppressive functional genes would be lower in soils used for intensive dairy production, compared with lower input pastoral soils (e.g. those used for expansive sheep grazing); and (3) variables associated with changes in the abundance of functional genes would be similar to those associated with the *Pseudomonas* community, but dissimilar to those associated with the total bacterial community.

## Materials and methods

### Soil sampling, physicochemical properties, and DNA extraction

Soil samples were collected from across the North and South Islands of New Zealand between November 2011 and January 2012, as part of the ‘New Zealand 50 Pastures Project’ described by Wakelin et al. [20]. Briefly, a broad survey of 50 New Zealand pasture soils was conducted covering 10 geographical regions and 11 of New Zealand’s major soil types (*sensu* New Zealand soil orders as defined by Hewitt [21]). Soils were sampled with full permission from the private land-owners for each site. No samples were collected without permission, nor from public conservation land.

From each of the 50 pasture sites, a single soil sample was collected. The aim of this work was not to determine variation within a field, farm, or catchment, but rather to assess changes across wider geographical ranges and with underlying soil properties. As such, the microbial community composition was compared within the soil type, nutrient status, and environmental properties associated with each individual site of collection, and comparisons made over the 50 pastures.

Approximately 2 kg of soil was collected at each site from a single sampling point, to a depth of approximately 15 cm [20], and stored at 4°C. Within 5 days of collection, DNA was extracted from 0.25 g of each of the 50 soils, in triplicate, using the PowerSoil DNA extraction kit (MoBio Inc, USA). Triplicate samples were pooled to increase total quantity of DNA available for analysis. The DNA concentration in each sample was quantified by spectrophotometry (ND-1000; ThermoFisher Inc).

For each soil, a comprehensive set of edaphic and environmental properties is available [20]. In addition, hot water extractable carbon (HWEC) and the aromatic content of the dissolved organic carbon (DOC) fraction were empirically measured. HWEC was extracted from 3 g (oven dry-weight equivalent) of field moist soil using the two-step process described by Ghani et al. [22]. HWEC solutions were filtered to 0.45 μm and analyzed by a Shimadzu 5000A TOC analyzer. Aliquots of the HWEC fraction were normalized by total dissolved organic carbon (DOC = total carbon–inorganic carbon) to a final concentration of 45 μg/ml before the aromatic component of the carbon (DOC aromatic content) was quantified by UV absorbance at 254 nm (FLUOstar Omega microplate reader, BMG Labtech; [23]). The complete abiotic dataset used for analysis in this study is given in S1 Table.

Analysis of the data utilised two broad approaches. The first utilised comparison of soil microbial communities among two groups of pastures: those used to support cow grazing for dairy production (pasture group 1 = Dairy), and ‘other’ grazing systems used to support sheep and/or beef grazing (pasture group 2 = Other). At the time of sampling, soils were divided into the two landuse groups, which are synonymous with intensification of grazing systems. In New Zealand’s farming systems, dairy-based grazing is typically highly intensified with inputs of nutrients, and often irrigation, to support continuous pasture production. For example, dairy farms in New Zealand typically use approximately 110 kg ha^-1^ of N fertiliser annually, compared with just 10 kg ha^-1^ N on sheep or beef farms [24]. The underlying differences between dairy and ‘other’ grazed systems, reflecting high or low system intensification, are reflected in a range of nutrients across the two groups [20]. For example, the concentrations of phosphorus, nitrogen and Sulphur (elements associated with fertilizer inputs) are significantly higher in dairy-based systems [20]. This grouped-based analysis aided in testing of H2. The second analysis approach assessed relationships between the soil microbial communities (phylogenetic and functional) and abiotic properties across the entire dataset. Here, it was not expected that variables associated with soil pedology, such as bulk density or Al concentrations, nor environmental variables, such as annual temperature, would be related to landuse or intensification. Analysis across the dataset allowed for testing of potential expression of associations between microbial communities and these variables. Hereon in, we classify soils to high or low farming intensity (dairy or other grazing systems), but use underlying nutrient levels as a measure of the continuum of intensification.

### Community (DNA) fingerprinting

#### Total bacteria: Terminal restriction fragment length polymorphism (TRFLP)

For bacterial community TRFLP analysis, primers 8F and 1520R ([25]; S2 Table) were fluorescently labelled at the 5ʹ and 3’ ends with FAM and HEX, respectively [16]. Each 25 μl reaction mixture contained 400 nM of each primer, 1 × Bioline MyHSTaq reaction buffer, 1 U MyHSTaq DNA Polymerase (BioLine Pty Ltd.), and 2 μl of template DNA (1:100 dilution of 10 ng/μl DNA). Thermocycling conditions are given in S2 Table. Reactions were validated by agarose gel electrophoresis. PCR products were digested (separate reactions), with AluI and CfoI (Promega) to generate fluor-labelled terminal restriction fragments (TRFs) of varying size. Pre- and post-digestion PCR products were purified using AxyPrep Mag PCR paramagnetic bead solution and 96 well magnetic plates, according to the manufacturer’s protocol.

Restriction fragments were separated by capillary electrophoresis (ABI 3730 DNA Analyzer) at the Australian Genome Research Facility (Adelaide, Australia). The lengths (base pairs) of individual TRFs were calculated by comparison to the internal size standard GS500LIZ (Applied Biosystems). Electropherograms were imported into Peak Scanner (Applied Biosystems), visually inspected for sizing quality and peak areas (in base pairs) determined for TRFs 50–500 bp in length. The online tool T-Rex [26] was then used to distinguish true peaks from background fluorescence [27]. The custom R script ‘interactive binner’ [28] provided bacterial community fingerprints by binning peaks to operational taxonomic units (OTUs). Each peak was inferred to be an OTU and the height of each peak used as a measure of the relative abundance of each OTU.

#### *Pseudomonas*: Denaturing gradient gel electrophoresis (DGGE)

A nested PCR approach was taken for the amplification of *Pseudomonas*-specific 16S rRNA gene fragments. Initially, *Pseudomonas*-specific PCR was conducted using primers F311Ps and R1459Ps ([29]; S2 Table). The presence of amplicons of the expected size was validated by agarose gel electrophoresis. Resultant PCR products were diluted 1/10 and used as template DNA for the second, general bacterial amplification using DGGE primers F968-GC and R1378 ([30]; S2 Table). Each 25 μl reaction mixture contained 200 nM of each primer, 1 × Bioline MyTaq reaction buffer, 1 U (*Pseudomonas*) or 0.625 U (Bacteria) MyHSTaq DNA Polymerase (BioLine Pty Ltd.), and 2 μl (20 ng) of template DNA. Thermocycling conditions (S2 Table) and DGGE methodology as described by Wakelin et al. [31]. The 50 PCR products were analyzed in a randomized order across three DGGE gels. Band location and intensity data were collected using TotalLab TL120 software (Nonlinear Dynamics, UK). Each band was inferred to be an OTU and band intensity data considered a measure of the abundance of each OTU.

Utilising a community approach, the primary focus of this study was to investigate relationships between indigenous bacterial and *Pseudomonas* communities, and edaphic, environmental, and farm-management factors. DNA fingerprinting techniques provide a useful exploratory approach to identify ecological patterns and, although affording lower taxonomic resolution than high throughput sequencing, have a similar capacity to correlate abiotic variables with separation in microbial community structure (β-diversity) [32, 33].

### Quantitative PCR (qPCR)

Quantitative PCR was used to assess the size of the bacterial and *Pseudomonas* communities in each DNA sample. Assays were conducted on a Rotor-Gene 6000 detection system (Qiagen). Bacteria-specific qPCR used primers Eub338 and Eub518 ([34]; S2 Table) with SYBR-Green detection. Each 25 μl reaction mixture contained 1 × SensiMix SYBR no-Rox master mix (BioLine Pty Ltd.), 500 nM of each primer, and 2 μl (20 ng) of template DNA. TaqMan-based qPCR was used to quantify *Pseudomonas*-specific 16S rRNA gene fragments. Primers Pse435F and Pse686R were used in combination with the dual-labelled hydrolysis probe Pse449 ([35]; S2 Table). Each 25 μl reaction mixture contained 1 × SensiMix II Probe master mix (BioLine Pty Ltd.), 300 nM of each primer, 150 nM probe and 2 μl (20 ng) template DNA. Thermocycling conditions for both reactions are detailed in S2 Table. Assay specificities were validated by melt curve analysis over a 50–95°C temperature gradient (SYBR-Green) or agarose gel electrophoresis (TaqMan).

The copy numbers of each gene were quantified against a standard curve that related standards of defined DNA concentration with threshold cycle (C_T_) values. Standard curves were generated from 10-fold serial dilutions of plasmid DNA containing the gene fragments of interest with five standard concentrations run in triplicate per assay. The target gene regions were amplified from DNA extracted from either soil or a reference strain (*Pseudomonas fluorescens* F113; AgResearch culture collection no. 1911) and PCR products cloned using the TOPO-TA cloning vector (Invitrogen). Samples were analyzed in triplicate across three machine runs. To account for run-to-run variation, an inter-run calibrator [36] was included in triplicate per run and standard curves adjusted accordingly.

Copy numbers were expressed per gram soil and the relative abundance of *Pseudomonas* spp. was calculated as the ratio between the group-specific qPCR assay and the bacteria-specific qPCR assay. The log_10_ values of bacteria copy numbers and *Pseudomonas*:bacteria ratio were used for statistical analysis. The relationship between standard concentrations and C_T_ values was linear for both qPCR assays across machine runs (R^2^>0.99) and amplification efficiency was consistently above 93%.

### GeoChip analysis of functional genes

The composition and abundance of functional genes were determined using the functional gene microarray, GeoChip 5.0 [37]. This is the first generation of the GeoChip to contain a suite of probes for the detection of secondary metabolism genes putatively associated with soil disease suppression. These include: antibiotic biosynthesis genes commonly associated with biocontrol Pseudomonads, *phlD* (2,4-diacetylphloroglucinol), *phzF* (phenazine), and *prnD* (pyrolnitrin); antibiotic biosynthesis genes of non-*Pseudomonas* bacteria, such as *bacA* (bacilysin) and *strR* (streptomycin); and genes required for the biosynthesis of the volatile compound hydrogen cyanide (*hcnB*). Alongside gene sets associated with lytic enzyme and siderophore production, these constitute a subset of 2002 probes (from the 167,044 on the array) that provide coverage of genes with a putative role in the suppression of soil-borne plant pathogens (S3 Table). The selected genes were characterized into one of three categories: (i) carbon degradation (chitinase and acetylglucosaminidase); (ii) nutrient competition (bacterial, fungal and archaeal siderophore production); and (iii) secondary metabolism (antibiotic biosynthesis genes).

Sample DNA (500 ng) was labelled by random priming with the fluorescent dye cyanine 3 and hybridized with the array as previously described ([37]; Institute for Environmental Genomics, University of Oklahoma). Raw data was pre-processed using an established microarray analysis pipeline (http://ieg.ou.edu/microarray/) as described by He et al. [38]. Poor-quality spots (signal-noise ratio < 2.0) were removed from the analysis and the signal intensity of each spot was normalized (divided by the total intensity of the microarray and multiplied by the average signal intensity of the microarray), prior to log-transformation of the data.

The gene categories defined above were used collectively (All Genes) and individually to assess the influence of soil properties on functional gene composition among soils (general analysis approach described in [39]). To compare the abundance of individual genes among soil samples, signal intensities were first standardized by the number of probes per gene (to account for disproportionate numbers of probes) and expressed as per gram of soil. Based on correlations among individual genes, 13 gene categories were defined for univariate analysis of functional gene abundance. Chitinase and acetyl-glucosaminidase gene abundances were highly correlated (>0.98), thus the carbon degradation gene category was retained in the dataset. Similarly, bacterial, fungal and archaeal siderophore production gene abundances were highly correlated (>0.94), thus the nutrient competition gene category was retained in the dataset. Likewise, *phzF* (phenazine) and *phzA* (phenazine) were highly correlated (0.98), and therefore *phzF* was retained in the dataset. The genes *bacA* (bacilysin), *pabA* (chloramphenicol), *hcnB* (cyanide), *phlD* (DAPG), *lgrD* (gramicidin), *lmbA* (lincomycin), *prnD* (pyrolnitrin), *strR* (streptomycin), *spaR* (subtilin), and *pcbC* (β-lactam) gene abundances were not strongly correlated and were therefore analyzed as individual genes. The array contained only one probe for the *pltC* (pyoluteorin) and *prnB* (pyrolnitrin) genes; these were removed from the analysis. GeoChip data (2002 probes) for the 50 soils sampled are available in S3 Table. Furthermore, these data are archived in the NCBI GEO database, accession number GSE112489.

### Statistical analysis

#### Biogeography of bacterial and *Pseudomonas* communities across New Zealand pastoral soils

Relationships between geographic distance (km between sampling points) and soil microbial community composition were tested across the 50 pasture sites spanning New Zealand (total distance ~ 1,300 km). The underlying hypothesis is that if local soil type and/or environmental factors are associated with disease suppressive microbiota, then a link to geographical proximity would be evident in the dataset. However, if influences of management practices (intensification) were dominant, these would override/obscure biogeographical effects.

Pair-wise Euclidean distances between sampling points were calculated from the GPS coordinates. Similarly, ecological ‘distances’ in microbial community assemblages among soils were calculated using the Bray-Curtis method from biological TRFLP or DGGE OTU data (standardised and square root-transformed; [40]). Simple distance-decay relationships were tested among the geographic and biological distance matrices using non-parametric (Spearman’s; ρ) correlation with permutation (x 999) based generation of a null-distribution to enable probability-based confidence (RELATE test; [41]).

#### Influence of land use and soil type on bacterial and *Pseudomonas* communities, and disease suppressive functional genes

**Fig 1:**
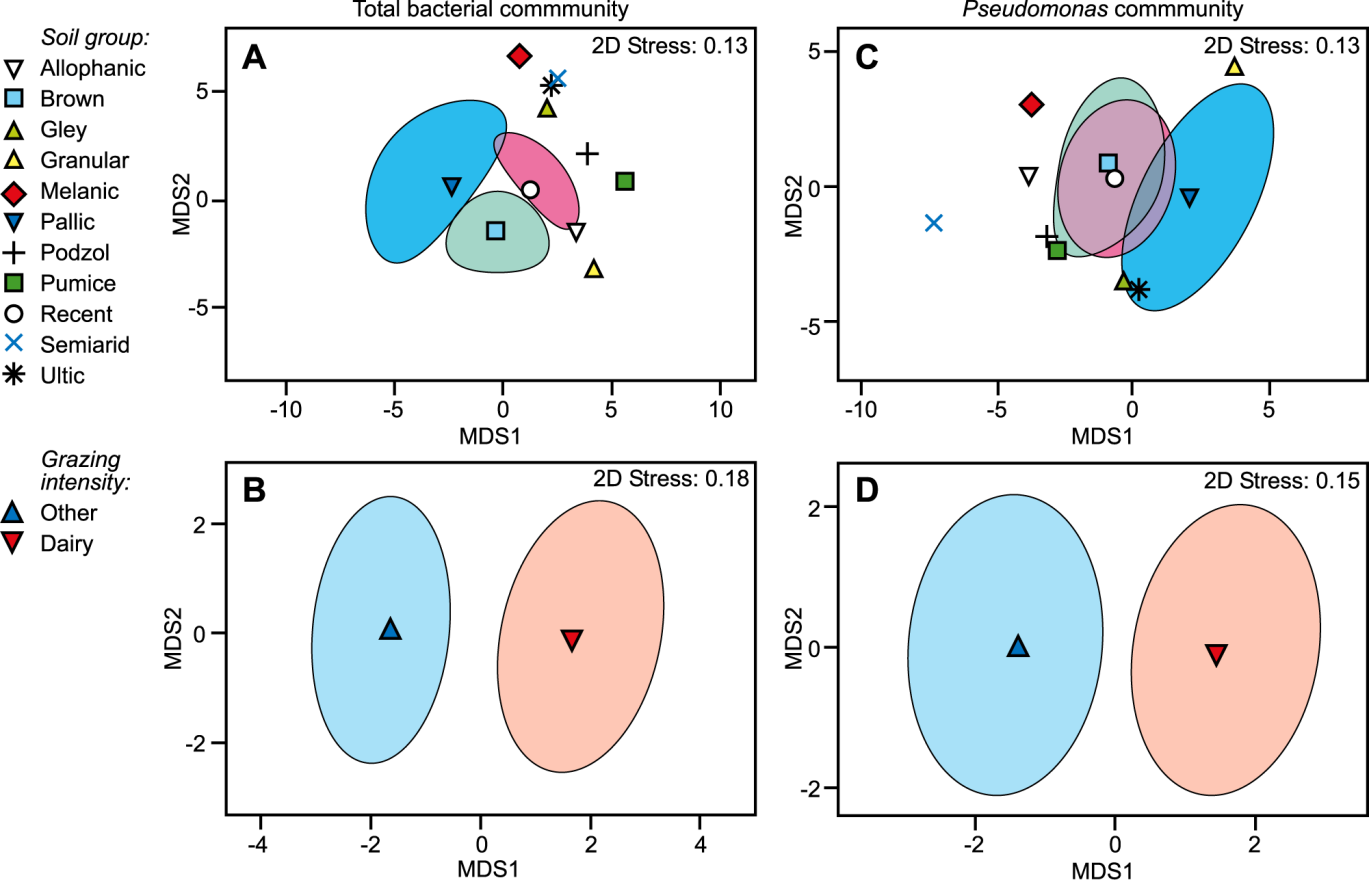
**Influence of soil type and land use on microbial community structure: metric MDS ordination plots of mean total bacterial (A and B) and *Pseudomonas* (C and D) communities.** The structures of the total bacterial and *Pseudomonas* communities were assessed based on the relative abundance of terminal restriction fragments (TRFs) and denaturing gradient gel electrophoresis (DGGE) banding patterns, respectively. Mean communities (individual points) for each land use and soil type were derived from 150 bootstrap averages. For land uses and soil types with sufficient replication, 95% region estimates for the mean communities (clouds) represent the spread of the bootstrap averages. Points and/or 95% region estimates in closer proximity represent groups that share increasing similarity in microbial community structure. Observations are statistically supported by PERMANOVA testing of Bray-Curtis dissimilarity data (S4 Table). Underlying OTU data for T-RFLP and DGGE analysis is available in S5 and S6 Tables, respectively.

Similarity in the composition of disease suppressive genes between soils was calculated from the sub-set of GeoChip data using the Bray-Curtis method (as above). From the respective distance matrices (bacterial, *Pseudomonas*, and functional genes), permutation-based multivariate analysis of variance (PERMANOVA; 999 permutations; [42]) was used to partition variance in composition associated with land use by comparing high (dairy) or low (other) farm system intensification/ landuse types, and soil type (11 New Zealand soil orders; Fig 1). Potential influences of sample distribution across gels (DGGE) on the structure of the *Pseudomonas* community were accounted for in the analysis; these were not significant (PERMANOVA; *P* = 0.302). These analyses were performed in PERMANOVA/PRIMER7 using described methods (PRIMER-E Ltd., UK; [43, 44]).

**Fig 2:**
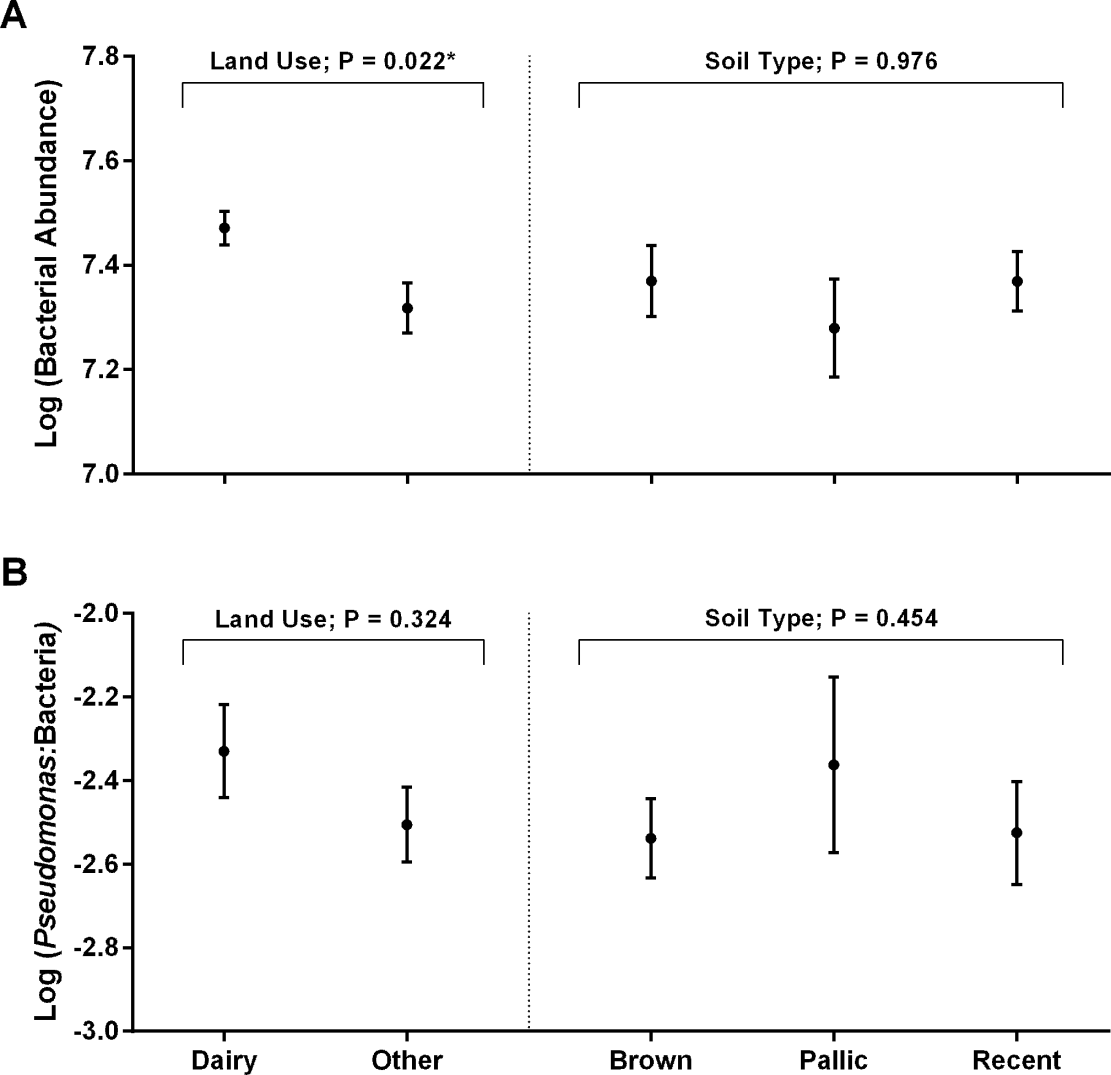
**Influence of land use and soil type on (A) the abundance of the total bacterial community and (B) the relative abundance of the *Pseudomonas* community (mean ± SEM).** The size of the total bacterial and *Pseudomonas* communities were determined by quantitative PCR. The effects of land use and soil type were formally tested by REML analysis (Genstat). Samples were characterized by land use as either high intensity ‘dairy’ systems or ‘other’, relatively lower intensity pasture systems, e.g. sheep and beef grazing systems.

Analyses of community size (microbial, qPCR, or functional gene, GeoChip abundance data) were performed in Genstat for Windows (17^th^ Edition). Residual maximum likelihood (REML) analysis of linear mixed models tested for effects of land use and soil type (three New Zealand soil orders; Fig 2). For these three soil orders (brown, recent, and pallic), sufficient sampling replication exists to assess soil-type influences on abundance following a univariate analysis approach.

#### Linking edaphic and environmental properties to the composition and abundance of microbial communities and functional genes

To reduce the size of the edaphic and environmental (abiotic) dataset, a correlation matrix was generated and all but one of a highly mutually correlated (>90%) set of variables were removed from the analysis. Skewed abiotic variables were transformed to correct the distribution, and all abiotic variables were normalized to obtain homogeneous variances [44]. The transformed and normalized dataset was applied in both multi- and univariate analysis of the data.

BIOENV analysis (biota and/or environment matching; [45]) was used to find the highest rank correlation (ρ) between the community assemblage data (Bray-Curtis Matrices) and the associated soil and environmental variables (Euclidian Distance Matrix). The rank correlation (ρ) indicates the amount of variation in the assemblage data that can be explained by the BIOENV-selected abiotic variables. BIOENV was optimized for four variables and *P* values derived from non-parametric Mantel-type testing (99 permutations; BIO-ENV, PRIMER).

Step-wise regression analysis was used to select the five abiotic variables that collectively explained the most variation in abundance data. ‘Total bacteria’, as determined by qPCR analysis in this study, was added to the abiotic variables for regression analysis of functional gene abundances (GeoChip).

For all statistical analyses, *P* values were considered significant when ≤0.05 and marginally significant when between 0.05 and 0.10.

### Functional molecular ecological network (fMEN) analysis

**Fig 3:**
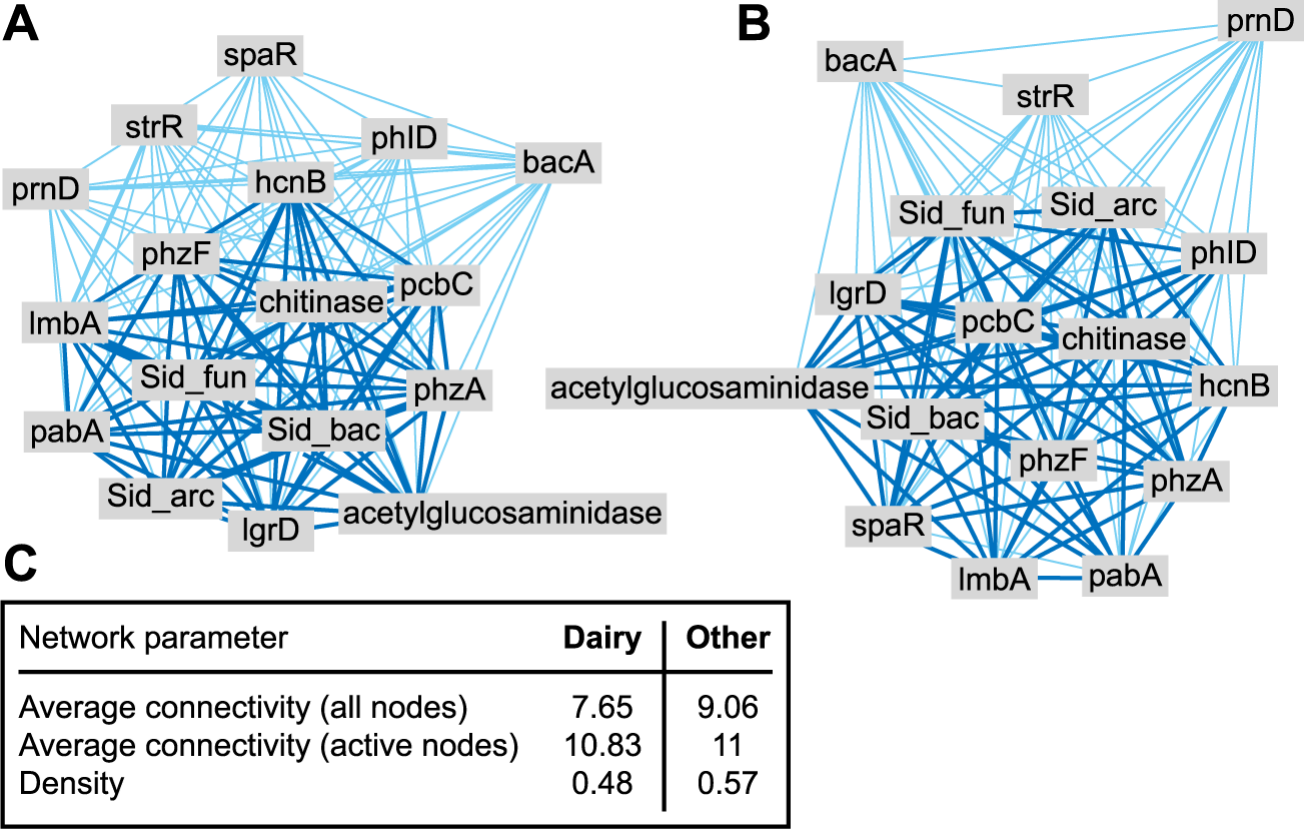
**Network plots of disease suppressive functional gene abundance in (A) dairy (high intensity) and (B) other (low intensity) pasture soil.** Nodes represent each individual gene, rather than gene categories, with a putative role in disease suppression. Edges (blue lines) correspond to associations between genes; bold lines reflect Pearson correlations ≥ 0.9, and fine lines correlations between 0.8 and 0.9. Sid_fun, Sid_arc and Sid_bac represent fungal, archaeal and bacterial siderophore production genes, respectively. (C) Measures of average connectivity and density were higher for other (low intensity) systems than for dairy (high intensity) systems.

To assess co-occurrence patterns, abundance data of individual functional genes (derived from GeoChip hybridization intensity data) was used to generate separate correlation-based networks for high (dairy) and low (other) intensity pasture systems (Fig 3). Nodes in the network represent individual functional genes (labelled as such) and edges, or connections, between nodes represent the strength of the correlation between nodes. Networks were derived from Pearson correlation matrices and connection edges above the threshold of 0.9 are represented by bold lines, while connection edges between 0.8 and 0.9 are represented by fine lines. The appropriate threshold was selected using the random matrix theory (RMT)-based network approach [46] applied to the entire GeoChip dataset. Cytoscape 3.3.0 software was used to visualize networks and network parameters average connectivity and density [46] were calculated to describe differences between network structures.

## Results

### Bacterial and *Pseudomonas* community structure and abundance

Soil bacterial community structure was assessed based on the relative abundance of terminal restriction fragments (TRFs; total bacteria) and denaturing gradient gel electrophoresis (DGGE) banding patterns (*Pseudomonas*). Furthermore, the sizes of the bacterial and *Pseudomonas* communities were determined by qPCR analysis. Across the 50 soils, total bacterial 16S rRNA gene abundance was 4.24 × 10^7^ (± 3.78 × 10^5^) per gram of soil, and the *Pseudomonas* community represented less than 1% of this total bacterial community.

There was a distinct lack of association between geographic distance and either total bacteria (*P* = 0.681) or *Pseudomonas* (*P* = 0.408) community structures. Furthermore, when the analysis was constrained to a single soil type (e.g. within Brown soils) or within a land use system (e.g. dairy), no biogeographical influences were present.

The results of PERMANOVA analyses that partitioned the influences of land use and soil type on bacterial and *Pseudomonas* community composition showed that land use impacted the structure of both the total bacterial (*P* = 0.035) and *Pseudomonas* communities (*P* = 0.084) (S4 Table). However, microbial community composition had no relationship to soil type (*P*>0.97). These influences were evident in the MDS ordination (Fig 1). Clear separation of microbial community structures by land use was evident (Fig 1B and 1D), however there was no partitioning of community similarities by soil type (Fig 1A and 1C). It is important to note that a large proportion of the variation in community composition among samples could not be explained by either land use or soil type (residual √CV; S4 Table).

As determined by REML analyses, land use had a significant effect on the size of the bacterial community, with higher numbers of bacteria in soils under dairy pasture compared with those under ‘other’ (lower intensity) pasture systems (Fig 2A). In contrast, the influence of land use intensity on *Pseudomonas* relative abundance was not significant (Fig 2B). Soil type had no effect on either the abundance of bacteria or relative abundance of *Pseudomonas* spp. (Fig 2).

### Linking edaphic and environmental properties to bacterial and *Pseudomonas* communities

The extent to which the variation in microbial community composition could be explained by soil and environmental properties was determined by BIOENV analysis (Table 1). No correlation between the total bacterial community structure and abiotic variables was evident when the analysis was optimized for four variables. However, assessment of individual correlations revealed that variation in pH (5.0–6.8) explained close to 20% of the variation in the structure of the bacterial community (ρ = 0.195; *P* = 0.06).

**Table 1:** Edaphic and environmental properties influencing microbial community structure.

BIOENV Analysis	Bacteria	*Pseudomonas*		
Spearman rank correlation (ρ^a^)	26%	37.1%		
P^b^	0.542	0.059		
**Edaphic & environmental variables**			**ρ**^**c**^	**P**^b^
		Sodium	22%	0.03
		C:P Ratio	15%	0.028
		pH	10%	0.083
		Rainfall	9%	0.122

^a^BIOENV analysis was used to find the highest rank correlation between the bacteria (TRFLP) or *Pseudomonas* (DGGE) community assemblage data and the associated soil and environmental variables. Spearman rank correlations (ρ) indicate percentage variation accounted for by the selected variables. ρ^a^ was optimized for four edaphic and environmental variables.

^b^P values were derived from permutation testing (× 999).

^c^For *Pseudomonas* the correlation was significant and these variables are listed in order of decreasing individual correlations (ρ^b^; RELATE-test).

In contrast, 37% of the variation in *Pseudomonas* community profiles between samples was associated with variation in four soil and environmental properties (Table 1). Soil sodium content was associated with the greatest proportion of this variation (22%). The quality of soil organic matter, rather than the quantity (i.e. carbon availability *sensu* C:P ratio), also had a significant correlation (15%). Individual correlations of *Pseudomonas* community assemblage data with C:N ratio (ρ = 0.131, *P* = 0.081) and DOC aromatic content (ρ = 0.121, *P* = 0.084) supported this finding. The influence of pH on *Pseudomonas* spp. composition (10%) was substantially reduced in comparison to the total bacterial community (19.5%).

Although total bacterial abundance and *Pseudomonas* relative abundance were correlated (*P* = 0.019), the variance accounted for by the regression was only 9.2% (data not shown). To identify which abiotic variables influenced microbial community abundances, step-wise regression analysis was performed (Table 2). With the exception of potassium (selected as the third parameter in both models), the explanatory variables differed between the total bacterial and *Pseudomonas* communities. Soil temperature and DOC aromatic content were the first parameters selected to explain total bacterial and *Pseudomonas* relative abundance, respectively (Table 2). Soil sodium content was the only variable to be negatively correlated with bacterial abundance. DOC aromatic content was positively associated with *Pseudomonas* relative abundance (*P* = 0.003; Table 2). Individual linear regressions attributed 15.6% and 13.5% of the variation in *Pseudomonas* relative abundance to DOC aromatic content and volume weight, respectively.

**Table 2:** Edaphic and environmental properties influencing microbial community abundance.

	Bacteria abundance			*Pseudomonas* relative abundance		
Significance of regression (P)	<0.001			0.007		
R^2^	51.8%			30.3%		
Regression model terms^a^		P^b^	Slope^c^		P^b^	Slope^c^
**1**^**st**^	Soil temperature	<0.001	0.085	DOC Aromatic Content	0.003	0.082
**2**^**nd**^	Sodium	0.002	-0.142	Volume weight^d^	0.129	0.199
**3**^**rd**^	Potassium	<0.001	0.064	Potassium	0.119	0.136
**4**^**th**^	Iron	0.045	0.061	Rainfall	0.16	0.132
**5**^**th**^	Sulphate sulphur	0.067	0.050	Total Calcium	0.155	0.100

^a^Step-wise regression analysis selected the five variables that collectively explained the most variation in the community abundance (qPCR). Terms of the regression model are listed in the order they were added to the model.

^b^*P* values were derived from accumulated analysis of variance.

^c^Slopes of individual regressions describe the relationship between abundance and variables in the regression model.

^**d**^Volume weight is an indicator of soil bulk density.

### Composition and abundance of disease suppressive functional genes

Multivariate analyses of three GeoChip gene categories (carbon degradation, nutrient competition, and secondary metabolism) showed no evidence of biogeographical separation (all *P* values >0.114) or influence of soil type (*P*>0.27; Table 3) on functional gene composition. Variation in land use, however, was found to be associated with each of the defined gene-categories (*P*<0.063), and there was support for a soil type × land use effect (*P*<0.097; Table 3). The influence of land use was not associated with the environmental or physicochemical soil properties measured in the study. For each of the gene categories, the correlation between functional gene composition and the soil and environmental variables was weak (BIOENV results; Table 3).

**Table 3:** Influence of soil type, land use and abiotic properties on the composition of disease suppressive genes.

	All Genes		Carbon Degradation		Nutrient Competition		Secondary Metabolism	
**PERMANOVA**^**a**^	**√CV**^**c**^	**P**^**d**^	**√CV**	**P**	**√CV**	**P**	**√CV**	**P**
Soil Type	1.56	0.315	1.78	0.274	0.29	0.43	0.78	0.395
Land Use	4.25	**0.049**	4.48	**0.056**	3.57	**0.063**	3.52	**0.051**
Soil Type x Land Use	3.56	**0.097**	3.75	0.113	3.59	**0.084**	2.67	0.137
Residual	7.91		8.23		7.51		6.7	
**BIOENV**^**b**^								
Spearman Rank Correlation (ρ)	0.202		0.203		0.206		0.194	
P^b^	0.72		0.76		0.76		0.83	

^a^PERMANOVA tested for effects of soil type and land use (‘dairy’ or ‘other’) on functional gene composition.

^b^BIOENV analysis was used to identify soil and environmental variables accounting for the variation (ρ) in functional gene composition.

^c^√CV is the square-root of the component of variation (Anderson et al. 2008),[43], which provides a measure of the size of effect for each component in the analysis.

^d^P values for both analyses were derived from permutation testing (x999; PERMANOVA and BIOENV, PRIMER).

The abundances of 13 individual functional genes/gene categories (see materials and methods) were assessed by univariate analysis. There was no effect of land use or soil type on functional gene abundance (REML; data not shown). To determine shared drivers of disease suppressive gene abundance, step-wise regression models were generated for each of the genes/gene categories (S7 Table). The five biotic and abiotic soil properties most commonly included in individual regression models were fitted as a reduced linear model (Table 4). Regressions with this reduced model were significant (*P*<0.05) for all but one of the suppressive genes/gene categories, and explained between 17% and 61% of the variation in gene abundances (Table 4).

**Table 4:** The variation in disease suppressive gene abundance accounted for by a reduced linear model.

	Disease suppressive genes												
	CD^b^	*bacA*	*pabA*	*hcnB*	*phlD*	*lgrD*	*spaR*	*pcbC*	*lmbA*	*phzF*	*prnD*	*strR*	NC^c^
**P Value**	**< .001**	**0.309**	**<0.001**	**<0.001**	**0.002**	**<0.001**	**<0.001**	**<0.001**	**<0.001**	**<0.001**	**0.028**	**0.004**	**<0.001**
**% Variance accounted for by model (R**^**2**^**)**	**49.8**	**2.6**	**43.5**	**52.1**	**29.6**	**52**	**42.1**	**49.2**	**61**	**54.2**	**17**	**26.2**	**53.3**
**Reduced Fitted Model**^**a**^**: **													
Total bacteria	0.030	0.014	0.017	0.042	0.016	0.013	0.002	0.032	0.050	0.042	0.068	0.030	0.036
Total carbon^d^	0.010	0.000	0.009	0.015	0.014	0.010	0.005	0.012	0.015	0.012	0.000	0.013	0.012
Extractable aluminium	-0.013	-0.003	-0.005	-0.018	-0.008	-0.011	-0.007	-0.013	-0.021	-0.016	-0.023	-0.011	-0.015
DOC aromatic content	-0.012	-0.007	-0.006	-0.011	-0.008	-0.008	-0.004	-0.012	-0.017	-0.014	-0.025	-0.015	-0.014
Soil moisture	0.009	0.003	0.004	0.010	0.005	0.010	0.004	0.011	0.021	0.013	0.003	0.013	0.012

^a^The reduced linear model was derived from the five biotic and abiotic variables that most commonly occurred in step-wise regression models generated for the abundance of 13 individual genes or gene categories. The reduced model was fitted by generalized linear model regression analysis. Numbers associated with each variable and functional gene/gene category are the slopes of individual regressions and describe the relationship between a particular variable and the abundance of that gene.

^b^Carbon degradation (CD).

^c^Nutrient competition (NC).

^d^Total carbon is representative of total carbon, organic matter content, total nitrogen and total sulphur (see materials and methods)

The size of the total bacterial community, total carbon (representative of total carbon, organic matter content, total nitrogen and total sulphur), DOC aromatic content, and extractable aluminum were associated with abundances of disease suppressive genes, with each of these variables included in eight or more of the 13 original regression models (S7 Table). In all models, functional gene abundance increased with increasing bacterial abundance, total carbon and soil moisture, and decreased with increasing DOC aromatic content and extractable aluminum (Table 4).

Based on functional gene abundance data, network analysis was used to assess associations among disease suppressive genes from both dairy and other pasture systems (Fig 3). In the low farming-intensity network, the abundance of three out of 17 functional genes had relatively weak correlation (≥0.9) with the other genes (Other; Fig 3B). Whereas, five of the 17 functional genes did not correlate (≥0.9) with the abundance of any other genes in the high intensity network (Dairy; Fig 3A). Furthermore, differences in network structure between land uses were observed; the network parameters connectivity and density were higher in the low intensity (other) network than the high intensity (dairy) network (Fig 3C).

## Discussion

### Land use and soil chemical properties are key drivers of total bacteria and *Pseudomonas* communities

Land use intensification was identified as a key factor associated with variation in the structure of both total bacterial and *Pseudomonas* communities, and also the abundance of bacteria. Soil properties that differed between land uses in these soils were primarily associated with fertilizer inputs (phosphorus, nitrogen and sulphur; [20]), and carbon recalcitrance (DOC aromatic carbon; this study). Although these properties were associated with alteration of *Pseudomonas* community composition and variation in bacterial abundance, they were not linked to variation in bacterial community structure.

In agreement with multiple observations across local, regional and continental scales [47–49], variation in soil pH was the primary variable associated with bacterial community composition. Soil pH did not differ between land uses, and explained 20% of the variation in bacterial community structure. As such, a large proportion of the remaining variation was unaccounted for and likely attributable to factors not directly measured in our study, for example stocking rate or the botanical composition of the pastures.

*Pseudomonas* bacteria were used as an indicator group for putative disease suppressive potential in these pasture soils [5, 6]. The structure of the *Pseudomonas* communities in soils varied with soil organic matter quality (C:P ratio; nutrient stoichiometry), a finding directly supported by previous studies conducted under long-term, controlled, field-based manipulation of P inputs [31]. In the current study, the C content in the soils was similar between land-uses [20]; as such, variation in C:P ratio was most likely driven by higher mineral fertilizer inputs in the high-input (dairy) systems. Similarly, the abundance of *Pseudomonas* bacteria was correlated with the aromaticity of the dissolved organic carbon (DOC) content of the soil, a finding supported by studies in which *Pseudomonas* communities were shown to be sensitive to the soil DOC fraction under grass-clover leys [50]. These findings may have important implications for the potential to manage populations of these bacteria through alteration of soil organic matter quality. This may be achieved, for example, by managing the timing and magnitude of fertilizer inputs, or through inputs of farm-yard manure or plant residue management [31, 51].

There was no influence of biogeographic (spatial) distance on the structure of the bacterial community in these New Zealand pastoral soils. This finding is important as it indicates that factors that vary on the scales studied, particularly environmental conditions or regional soil types, are unlikely to be important, first-order drivers of the general soil microbial communities in managed pastoral ecosystems. These findings are in broad agreement with other studies of soil bacterial biogeography across landscapes spanning a similar range in latitude to our study (~1300 km) [52].

The finding that total bacterial and *Pseudomonas* community size and composition did not vary with soil type is in contrast to previous studies where significant influences of soil type on microbial communities have been found in both bulk [53] and rhizosphere soil [54]. This may reflect that while soils in this study were selected to represent a range of New Zealand soil orders, they were all collected from under pastoral agriculture where soil properties had been managed to optimize plant production. It is, therefore, not surprising that the inputs into these systems, as reflected in soil chemistry, were of greater importance than soil pedogenesis in structuring soil communities.

Collectively, these findings demonstrate that there are some common factors influencing variation in both total bacterial and *Pseudomonas* communities (land use and specific soil properties, such as pH), on this basis, H1 was rejected. However, factors associated with variation in *Pseudomonas* communities alone, notably soil organic matter quality, can be considered ‘specific’ to this taxa.

### Land use intensification impacts on disease suppressive functional gene composition and microbial community networks

Land use intensification was identified as a key factor influencing the composition of all putative disease suppressive functional gene categories. The impact of agricultural management on functional gene composition was not driven through changes in the soil properties measured in this study but as our study is the first to focus specifically on putative disease suppressive functional genes using GeoChip, comparable studies that target a range of disease suppressive genes are required to verify such findings across a broader range of land uses.

Ecological networks based on functional gene abundance data provide a means of visualizing interactions among members of the microbial community carrying these functional genes, and how these interactions differ between farming systems [55]. The abundance of individual genes did not differ significantly between land uses (H2 was rejected) but network analysis revealed associations (co-variance) among these genes were affected by land use. Further, it is interesting to note that the genes which fall outside of the ≥0.9 correlation threshold in both networks are also those for which the explanatory model of soil properties was either insignificant or explained a relatively low proportion of the variation in abundance (Table 3).

The greater connectivity and density of the ‘other’ farming system network, in comparison to that of the dairy farming systems, indicate that the structure of the network and interactions among functional groups are more complex under lower intensity pasture. Increased complexity of network structures may reflect stronger coupling or association of processes within the soil community, potentially contributing to greater stability of function [56]. Network analysis is an emergent procedure and provides a ‘conceptual framework’ that requires further experimental validation [56]. However, the sensitivity of functional molecular ecological network structure to variation in land use observed here was also revealed in response to varied land cover (forest succession; [56]) and environmental change (elevated CO2; [55]). Collectively, our results suggest that, in addition to impacting upon microbial community structure (Fig 1) and functional composition (Table 3), land use also affected the associations among these communities.

### Shared drivers of disease suppressive functional gene abundance

The reduced linear model of biotic and abiotic soil properties explained up to 61% of the variation in the abundance of 12 of the 13 putative disease suppressive functional genes/gene categories studied (Table 3). As suppressive modes of action are not mutually exclusive, multiple antagonists with a range of these functions may act together to suppress disease [57]. As such, the explanatory model was comprised of five variables shared among models generated for individual genes/gene categories with putative roles in carbon degradation (hyperparasitism), nutrient competition (siderophore production), and antibiosis (secondary metabolite biosynthesis). Although the distribution or occurrence of these functional genes under pastoral agriculture has been vastly understudied [2], the abundance of microbes associated with these functions has been shown to positively correlate with disease suppression in arable and natural grassland soils [13, 17–19, 58, 59].The biotic and edaphic properties positively correlated with abundance of these functional genes (Table 3) present opportunities by which the disease suppressive potential of soils may be managed and enhanced in pastoral systems. It will be important to validate associations between functional gene abundance and soil disease suppressiveness in pastoral ecosystems.

The abundances of all putative disease-suppressive functional genes/gene categories were positively correlated with the size of the bacterial community (total bacteria), total carbon, and soil moisture. It is possible that the inclusion of bacterial abundance in this model reflects the large proportion of the selected probes that were designed based on sequences of bacterial origin (S3 Table); probe design remains a limitation of functional gene microarrays [38]. However, the model was significant (and the relationship with total bacteria the same) for gene categories that also contained fungal and archaeal probe sequences (i.e. carbon degradation and nutrient competition). Therefore, it is likely that the inclusion of bacterial abundance in the model is also indicative of an influence of variation in the wider microbial community, for example, microbial biomass or fungal:bacterial ratios.

Our results suggest that practices that enhance the size of the bacterial community may also enhance the size of the disease suppressive community in pastoral soils. The additional incorporation of abiotic properties total carbon (representative of organic matter, total N and total S; see materials and methods) and soil moisture in the explanatory model is likely reflective of the dependence of general variables, such as microbial abundance, on nutrient availability and organic matter content [60]. Enhancing microbial abundance and activity through the addition of organic matter to soil is often associated with increased suppressiveness toward soil-borne plant pathogens [61]. Notably, this mechanism is typically referred to as general suppression [61], the enhancement of which has been identified as a potential opportunity to manage diverse soil-borne diseases in pasture systems [2].

The abundances of all putative disease suppressive genes/gene categories were negatively correlated with DOC aromatic content, a measure of carbon recalcitrance. This suggests that, in addition to organic matter quantity (total carbon), the abundance of these functional genes is sensitive to soil organic matter (SOM) quality and could be enhanced under copiotrophic conditions where labile carbon and nutrient availability is increased. The similarity in variables associated with functional gene abundance and *Pseudomonas* community composition, notably SOM quality, provides support for our hypothesis that there would be common drivers of these components of the soil microbial community (H3). Our study provides novel evidence for the association of SOM quality with a wide array of functional genes with putative disease suppressive function. This may provide mechanistic insights into the reduction of disease severity associated with suppressive organic amendments that support copiotrophic communities.

Despite inherent limitations (lack of microbial species identity and failure to distinguish between active and inactive microbial cells; [15]), the GeoChip array provides insight into the presence of and diversity within known biological functions, and therefore, a way of linking microbial diversity to ecosystem processes and functions [38]. However, as changes at the gene or genus levels may not translate to changes in ecosystem processes [62], it will be important to rigorously test the relationship between the abiotic drivers identified here and disease suppressive function.

## Conclusions

This study has demonstrated the substantial effects of land use intensification and soil properties on both the structure and function of microbial communities putatively associated with soil suppressiveness. Independent of the total bacterial community, soil properties were identified as unique drivers of the *Pseudomonas* community, a potential taxonomic indicator of disease suppressive potential. Furthermore, our results suggest that the manipulation of abiotic soil properties related to the size and activity of the microbial community may simultaneously enhance the abundance of a wide array of disease suppressive functions in soil. Importantly, soil organic matter quality was shown to influence both the taxonomic (*Pseudomonas*) and functional disease suppressive components of soil. Collectively, our results provide opportunities by which these soil components may be managed on-farm.

The following hypotheses were derived from this study: (1) *Pseudomonas* species composition and function are sensitive to pastoral management-induced changes in SOM quantity and quality, e.g. via plant residue management; and (2) management-induced changes in *Pseudomonas* community structure and function will provide microbial predictors of soil suppressiveness. Detailed experiments that combine microbiome characterization and plant-pathogen bioassays are now required to test these hypotheses, thereby determining whether *Pseudomonas* communities and functional gene abundances provide accurate microbial predictors of disease suppressive potential in these soils. Such experiments would determine whether specific soil microbial community manipulation, stemming from dedicated management practices, translate to changes in disease suppressive potential and ultimately pastoral production.

## Supporting information

S1 Table Physico-chemical and environmental properties of 50 New Zealand pasture soils. (XLSX)

S2 Table PCR primers and thermocycling conditions used for assessing the structure and size of total bacterial and *Pseudomonas* communities in soil DNA. (DOCX)

S3 Table GeoChip 5.0: Probes that provide coverage of genes with a putative role in the suppression of soil-borne plant pathogens. (XLSX)

S4 Table Influence of soil type and land use on microbial community structure. (DOCX)

S5 Table Total bacteria (T-RFLP) OTU data. (XLSX)

S6 Table *Pseudomonas* (DGGE) OTU data. (XLSX)

S7 Table Individual step-wise regression models for each disease suppressive gene/gene category. (DOCX)
