# Supplementary material for: Effect of land use and soil organic matter quality on the structure and function of microbial communities in pastoral soils: Implications for disease suppression
Source: PLoS One. 2018 May 7;13(5):e0196581. doi: 10.1371/journal.pone.0196581 (PMC5937765; doi:10.1371/journal.pone.0196581)
Supplement: S2 Table — (DOCX) [file pone.0196581.s002.docx]

**S2_Table.** PCR primers and thermocycling conditions used for assessing the structure and size of total bacterial and *Pseudomonas* communities in soil DNA

| **Molecular application** | **Primer** | **Sequence (5’ – 3’)** | **Reference** | **Thermocycling conditions** |
| --- | --- | --- | --- | --- |
|  | | | | |
| *Pseudomonas* PCR-DGGE:  *Pseudomonas*-specific PCR | F311Ps  R1459Ps | CTGGTCTGAGAGGATGATCAGT  AATCACTCCGTGGTAACCGT | Milling et al. ([2005](#_ENREF_5)) | 95°C for 3min;  30 cycles of 95°C for 30s, 63°C for 60s and 72°C for 60s; 72°C for 10min. |
| *Pseudomonas* PCR-DGGE:  General bacteria PCR | F968-gc  R1378  gc | gc-AACGCGAAGAACCTTAC  CGGTGTGTACAAGGCCCGGGAACG  cgcccggggcgcgccccgggcggggcgggggcacgggggg | Heuer et al. ([1997](#_ENREF_3))  Nubel et al. ([1996](#_ENREF_7)) | 95°C for 3min;  25 cycles of 95°C for 15s, 56°C for 30s and 72°C for 30s; 72°C for 30min. |
| Bacteria T-RFLP | 8F  1520R | AGAGTTTGATCCTGGCTCAG  AAGGAGGTGATCCAGCCGCA | Weisburg et al. ([1991](#_ENREF_8))  Edwards et al. ([1989](#_ENREF_2)) | 95°C for 3min;  30 cycles of 95°C for 30s, 55°C for 30s and 72°C for 60s; 72°C for 10min. |
|  | | | | |
| Bacteria qPCR | Eub338  Eub518 | ACTCCTACGGGAGGCAGCAG  ATTACCGCGGCTGCTGG | Lane ([1991](#_ENREF_4))  Muyzer et al.([1993](#_ENREF_6)) | 95°C for 10min;  40 cycles of 95°C for 30s, 53°C for 30s and 72°C for 30s. |
| Pseudomonas qPCR | Pse435F  Pse686R  Pse449 (Probe) | ACTTTAAGTTGGGAGGAAGGG  ACACAGGAAATTCCACCACCC  Fam-ACAGAATAAGCACCGGCTAAC-BHQ | Bergmark et al. ([2012](#_ENREF_1)) | 95°C for 10min;  40 cycles of 95°C for 30s and 60°C for 60s. |

Bergmark, L., Poulsen, P.H.B., Al-Soud, W.A., Norman, A., Hansen, L.H., Sørensen, S.J., 2012. Assessment of the specificity of Burkholderia and Pseudomonas qPCR assays for detection of these genera in soil using 454 pyrosequencing. FEMS microbiology letters 333, 77-84.

Edwards, U., Rogall, T., Blöcker, H., Emde, M., Böttger, E.C., 1989. Isolation and direct complete nucleotide determination of entire genes. Characterization of a gene coding for 16S ribosomal RNA. Nucleic Acids Research 17, 7843-7853.

Heuer, H., Smalla, K., van Elsas, J.D., Trevors, J.T., Wellington, E.M., 1997. Application of denaturing gradient gel electrophoresis and temperature gradient gel electrophoresis for studying soil microbial communities. Modern soil microbiology., 353-373.

Lane, D., 1991. 16S/23S rRNA sequencing. In: Nucleic acid techniques in bacterial systematics. pp. 125-175.

Milling, A., Smalla, K., Maidl, F.X., Schloter, M., Munch, J.C., 2005. Effects of transgenic potatoes with an altered starch composition on the diversity of soil and rhizosphere bacteria and fungi. Plant and Soil 266, 23-39.

Muyzer, G., De Waal, E.C., Uitterlinden, A.G., 1993. Profiling of complex microbial populations by denaturing gradient gel electrophoresis analysis of polymerase chain reaction-amplified genes coding for 16S rRNA. Applied and environmental microbiology 59, 695-700.

Nübel, U., Engelen, B., Felske, A., Snaidr, J., Wieshuber, A., Amann, R.I., Ludwig, W., Backhaus, H., 1996. Sequence heterogeneities of genes encoding 16S rRNAs in Paenibacillus polymyxa detected by temperature gradient gel electrophoresis. Journal of bacteriology 178, 5636-5643.

Weisburg, W.G., Barns, S.M., Pelletier, D.A., Lane, D.J., 1991. 16S ribosomal DNA amplification for phylogenetic study. Journal of bacteriology 173, 697-703.
