# Supplementary material for: Effect of land use and soil organic matter quality on the structure and function of microbial communities in pastoral soils: Implications for disease suppression
Source: PLoS One. 2018 May 7;13(5):e0196581. doi: 10.1371/journal.pone.0196581 (PMC5937765; doi:10.1371/journal.pone.0196581)
Supplement: S4 Table — (DOCX) [file pone.0196581.s004.docx]

**S4 Table. Influence of soil type and land use on microbial community structure.**

|  | **Bacteria** | | ***Pseudomonas*** | |
| --- | --- | --- | --- | --- |
| **PERMANOVA** | **√CV** | **P** | **√CV** | **P** |
| Soil Type | -11.49 | 0.999 | -11.42 | 0.968 |
| Land Use | 8.6 | **0.035** | 7.96 | **0.084** |
| Residual | 40.27 |  | 35.59 |  |

Bray-Curtis similarity matrices of bacteria (T-RFLP) and Pseudomonas (DGGE) community assemblage data were analysed by permutation-based multivariate ANOVA. Soil type (11 soil orders) and land use (‘dairy’ or ‘other’) were fixed factors in the PERMANOVA design. √CV is the square-root of the component of variation (Anderson et al. 2008), which provides a measure of the size of effect for each component in the analysis. P values were derived from permutation testing (x999; PERMANOVA, PRIMER).

**Reference:** Anderson MJ, Gorley RN, Clarke KR. PERMANOVA + for PRIMER: Guide to Software and Statistical Methods. Plymouth, UK: PRIMER-R Ltd; 2008.
