# Supplementary material for: Effect of land use and soil organic matter quality on the structure and function of microbial communities in pastoral soils: Implications for disease suppression
Source: PLoS One. 2018 May 7;13(5):e0196581. doi: 10.1371/journal.pone.0196581 (PMC5937765; doi:10.1371/journal.pone.0196581)
Supplement: S7 Table — (DOCX) [file pone.0196581.s007.docx]

**S6 Table. Individual step-wise regression models for each disease suppressive gene/gene category.**

|  | **Disease suppressive genes** | | | | | | | | | | | | |
| --- | --- | --- | --- | --- | --- | --- | --- | --- | --- | --- | --- | --- | --- |
|  | **CC^a^** | **bacA** | **pabA** | **hcnB** | **phlD** | **lgrD** | **spaR** | **pcbC** | **lmbA** | **phzF** | **prnD** | **strR** | **NC^b^** |
| **P Value** | **<0.001** | **0.007** | **<0.001** | **<0.001** | **<0.001** | **<0.001** | **<0.001** | **<0.001** | **<0.001** | **<0.001** | **<0.001** | **<0.001** | **<0.001** |
| **% Variance accounted for by model (R^2^)** | **61.5** | **32.8** | **64** | **63.4** | **47.4** | **7.04** | **68.3** | **64.3** | **72.5** | **67** | **49.4** | **49.4** | **66.4** |
| **Soil Env Properties** |  | | | | | | | | | | | | |
|  |  |  |  |  |  |  |  |  |  |  |  |  |  |
| Total carbon | 0.0164 |  | 0.0068 | 0.0286 | 0.0185 |  |  | 0.0087 |  | 0.0088 |  | 0.0328 | 0.0089 |
| Total bacteria | 0.0734 | 0.0350 | 0.0518 | 0.0771 | 0.0606 | 0.0617 | 0.0290 | 0.0785 | 0.1188 | 0.0890 | 0.1873 | 0.0941 | 0.0820 |
| Zinc | -0.0068 | -0.0114 |  |  | -0.0119 |  |  |  |  |  | -0.0170 | -0.0230 |  |
| Extractable aluminium | -0.0121 |  |  | -0.0252 |  | -0.0075 | -0.0028 | -0.0123 | -0.0150 | -0.0149 |  |  | -0.0145 |
| DOC aromatic content | -0.0114 |  | -0.0076 | -0.0126 |  | -0.0098 |  | -0.0146 | -0.0188 | -0.0173 |  |  | -0.0172 |
| Total cobalt |  | 0.0052 |  |  |  |  |  |  |  |  | 0.0150 |  |  |
| Total cadmium |  | 0.0138 |  |  |  |  |  |  |  |  |  |  |  |
| CEC |  | -0.0051 |  |  |  |  |  |  |  |  |  |  |  |
| %_d |  |  | 0.0052 |  |  | 0.0125 |  | 0.0128 | 0.0239 | 0.0150 |  |  | 0.0145 |
| pH |  |  | 0.0043 |  |  |  |  |  |  |  |  |  |  |
| Magnesium |  |  |  | -0.0148 |  |  |  |  |  |  |  |  |  |
| Total phosphorus |  |  |  |  | 0.0111 |  |  |  |  |  | -0.0217 |  |  |
| Potassium |  |  |  |  | -0.0076 |  |  |  |  |  |  | -0.0151 |  |
| Anaerobically mineralisable N |  |  |  |  |  | 0.0071 | 0.0096 |  | 0.0119 |  |  |  |  |
| rad |  |  |  |  |  |  | 0.0040 |  |  |  |  |  |  |
| Manganese |  |  |  |  |  |  | -0.0048 |  |  |  |  |  |  |
| TC |  |  |  |  |  |  |  |  |  |  | 0.0272 |  |  |
| Iron |  |  |  |  |  |  |  |  |  |  |  | 0.0159 |  |

^a^CD: carbon degradation

^b^NC: nutrient competition
